# Phenotypic characterization and 16S rDNA identification of culturable non-obligate halophilic bacterial communities from a hypersaline lake, La Sal del Rey, in extreme South Texas (USA)

**DOI:** 10.1186/2046-9063-8-5

**Published:** 2012-02-02

**Authors:** Kristen Phillips, Frederic Zaidan, Omar R Elizondo, Kristine L Lowe

**Affiliations:** 1 Science Department, Riverside Middle School, San Benito, Texas USA; 2 Department of Biology, University of Texas - Pan American, Edinburg, Texas USA

## Abstract

**Background:**

La Sal del Rey ("the King's Salt") is one of several naturally-occurring salt lakes in Hidalgo County, Texas and is part of the Lower Rio Grande Valley National Wildlife Refuge. The research objective was to isolate and characterize halophilic microorganisms from La Sal del Rey. Water samples were collected from the lake and a small creek that feeds into the lake. Soil samples were collected from land adjacent to the water sample locations. Sample salinity was determined using a refractometer. Samples were diluted and cultured on a synthetic saline medium to grow halophilic bacteria. The density of halophiles was estimated by viable plate counts. A collection of isolates was selected, gram-stained, tested for catalase, and characterized using API 20E^® ^test strips. Isolates were putatively identified by sequencing the 16S rDNA. Carbon source utilization by the microbial community from each sample site was examined using EcoPlate™ assays and the carbon utilization total activity of the community was determined.

**Results:**

Results showed that salinity ranged from 4 parts per thousand (ppt) at the lake water source to 420 ppt in water samples taken just along the lake shore. The density of halophilic bacteria in water samples ranged from 1.2 × 10^2 ^- 5.2 × 10^3 ^colony forming units per ml (cfu ml^-1^) whereas the density in soil samples ranged from 4.0 × 10^5 ^- 2.5 × 10^6 ^colony forming units per gram (cfu g^-1^). In general, as salinity increased the density of the bacterial community decreased. Microbial communities from water and soil samples were able to utilize 12 - 31 carbon substrates. The greatest number of substrates utilized was by water-borne communities compared to soil-based communities, especially at lower salinities. The majority of bacteria isolated were gram-negative, catalase-positive, rods. Biochemical profiles constructed from API 20E^® ^test strips showed that bacterial isolates from low-salinity water samples (4 ppt) showed the greatest phenotypic diversity with regards to the types and number of positive tests from the strip. Isolates taken from water samples at the highest salinity (420 ppt) tended to be less diverse and have only a limited number of positive tests. Sequencing of 16S DNA displayed the presence of members of bacterial genera *Bacillus*, *Halomonas*, *Pseudomonas*, *Exiguobacterium *and others. The genus *Bacillus *was most commonly identified. None of the isolates were members of the Archaea probably due to dilution of salts in the samples.

**Conclusions:**

The La Sal del Rey ecosystem supports a robust and diverse bacterial community despite the high salinity of the lake and soil. However, salinity does appear to a limiting factor with regards to the density and diversity of the bacterial communities that inhabit the lake and surrounding area.

## Background

Halophilic microorganisms have been isolated from many environments such as salterns [1], cold seeps [2], fish sauce [3], salt mines [4], brine wells [5] and salt lakes [6-8], including lakes in Antarctica [9] (for reviews, see [10,11]). Halophily is observed in members of the Bacteria, Archaea, and Eukarya [10] and there is diversity amongst halophiles with regards to the concentration of salts, temperatures, pH conditions, and redox conditions that the organisms are adapted. Thermophilic, psychrophilic, mesophilic, alkaliphilic, and aerobic halophiles have been described [12,13], as well as anaerobic halophilic bacteria capable of denitrification and sulfate reduction [14]. Halophiles play a role in the biogeochemistry of carbon and phosphorus in saline environments [15] and some halophiles have been shown to degrade organic compounds, such as pesticides and crude oil [16-19], for potential use in bioremediation studies and applications. In addition to bioremediation, halophiles may also have potential uses as biocontrol agents against certain pathogenic fungi [20,21].

A high saline environment places osmotic stress on living cells making them vulnerable to dehydration. Moreover, cellular proteins, including enzymes, may denature (unfold) at high salt concentrations leading to decreased enzyme activity and DNA damage [11]. Halophiles employ different morphological, physiological, and genetic mechanisms to withstand the environmental conditions in which they live. These include increasing internal cellular concentrations of solutes such as potassium ions (K+) or amino acid derivatives [10], having cell walls that are stabilized by sodium ions (Na+), and having extra copies of essential genes located on large plasmids [11]. Redundant genes may allow for the rapid repair of DNA that may become damaged due to high salinity; gene redundancy and rapid DNA repair is observed in other bacteria that inhabit environments, such as arid and desiccated environments, that can cause DNA damage. Such bacteria excise damaged DNA and use non-damaged areas as templates for repair [[22] and references therein].

Halotolerant and halophilic organisms are able to efficiently exclude sodium ions from the cell interior. Eukaryotic halophiles have been shown to possess ATP-driven Na+ pumps which actively remove the ions from the cell [23]. In bacteria, sodium removal is accomplished by sodium-proton exchange via Na+/H+ antiports [24].

Many halophiles build up concentrations of compatible solutes in their cytoplasm to combat the osmotic pressure exerted by the environment. This can be accomplished by either pumping inorganic ions into the cell from the environment or by synthesizing or concentrating an organic solute [11]. Low molecular weight organic molecules such as glycerol, sugars, glutamate, proline, and ectoine are examples of compatible solutes [11]. Glycine betaine, a methylated glycine deriviative, is widely used by halophilic bacteria as a compatible solute [11].

La Sal del Rey ("the King's Salt") is one of several naturally-occurring salt lakes in Hidalgo County, Texas, USA and is part of the Lower Rio Grande Valley National Wildlife Refuge [25]. The geological source of the salt in La Sal del Rey is unknown but archeological evidence suggests that prehistoric persons, Native American tribes, and Spanish, Mexican and American settlers mined salt from the lake for use in food preservation and mineral trade [26,27]. A diversity of wildlife species live near the lake, including mammals, birds, reptiles, amphibians, arachnids and insects. A recent phylogenetic study conducted by Hollister et al. [28] showed that the microbial community of La Sal del Rey is diverse and is represented by at least 24 bacterial and 2 archaeal phyla. However, that study relied solely on non-culture methods such as 16S rDNA sequencing and quantitative (Q)-PCR, and sampled soils and sediments along a transect leading up to the lake shore. As a complement to that study, we isolated potential prokaryotic halophiles from water in La Sal del Rey and soils at the lake shore. We characterized the microorganisms using traditional morphological, physiological, and phenotypic techniques along with 16S rDNA identification and compared organisms isolated from different regions of the La Sal del Rey that had disparate salinities.

## Methods

### Sample Collection and Site Characteristics

**Figure 1:**
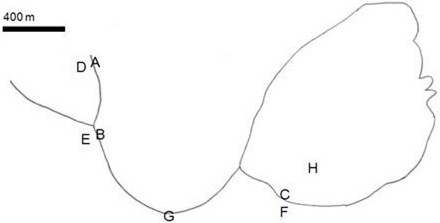
**Outline of La Sal del Rey with indications of sampling sites**. GPS coordinates of the salt lake are listed in the Methods section of the text. Bar = 400 m.

Water samples (5) and soil samples (3) were collected from locations in the La Sal del Rey area (26°31'55″ N, 98°03'50″ W; Figure 1). Sites A - F samples were taken on 14 June 2010; sites G - H samples were taken on 5 July 2010. Samples from A (water) and D (soil) were taken near the La Sal del Rey source water. Samples from B (water) and E (soil) were taken approximately 400 m south of the source. Samples from C (water) and F (soil) were taken at the lake shore, approximately 800 m east of sites B and E. Water was also collected from site G located mid-way between the water source and the lake and from within the lake (site H) after a major storm event, Hurricane Alex. Prior to the hurricane, the lake water level was visibly lower and the salinity of the water was higher.

Water samples were obtained by fully submersing a clean, sterile 50-mL plastic tube underneath the water surface. The lid was removed when the tube was 10-cm below the water surface to ensure that air was not introduced into the sample. The lid was replaced while submerged and the tube was appropriately labeled and dated. Soil samples were taken from the upper 10 cm using a small shovel and transferred to sterile containers. The samples were transported to the lab on ice and processed within 24 h of collection. Salinity of the water samples was measured using a model REF211ATC handheld refractometer (General Tools and Instruments, New York NY). Salinity of the soil samples was estimated by extracting porewater from the soil and measuring salinity using the handheld refractometer.

### Sample culturing

Serial dilutions (10^0^, 10^-1^, 10^-2^, and 10^-3^) of water and soil samples were made in sterile saline (0.85%). A 100 μL aliquot of each diluted sample was spread onto a saline media. The contents of the media per liter were peptone (5 g), yeast extract (1 g), ferric citrate (0.1 g), NaCl (19.45 g), MgCl_2 _(8.8 g), Na_2_SO_4 _(3.24 g), CaCl_2 _(1.8 g), KCl (0.55 g), NaHCO_3 _(0.16 g), KBr (0.08 g), SrCl_2 _(34.0 mg), BH_3_O_3 _(22.0 mg), Na_2_SiO_3 _(4.0 mg), NaF (2.4 mg), NH_4_NO_3 _(1.6 mg), Na_2_HPO_4 _(8.0 mg) and agar (15.0 g). Samples were plated in triplicate and incubated at 25°C for 7 d. Colonies growing on the plates were counted and the density of microorganisms in the original sample was estimated by multiplying the colony count times the dilution.

### Community Nutrient Profile

Water and soil samples collected were tested using BIOLOG EcoPlates™ (BIOLOG Inc., Hayward CA), which determine the types of carbon substrates that microbial communities can use. EcoPlates™ are 96-well microplates containing 31 different carbon sources in triplicate with a dye and 3 water (control) wells. The following carbon substrates were available on EcoPlates™: β-Methyl-D-glucoside, D-Galactonic Acid γ-Lactone, L-Arginine, Pyruvic Acid Methyl Ester, D-Xylose, D-Galacturonic Acid, L-Asparagine, Tween 40, i-Erythritol, 2-Hydroxy Benzoic Acid, L-Phenylalanine, Tween 80, D-Mannitol, 4-Hydroxy Benzoic Acid, L-Serine, α-Cyclodextrin, N-Acetyl-D-Glucosamine, γ-Hydroxybutyric Acid, L-Threonine, Glycogen, D-Glucosaminic Acid, Itaconic Acid, L-Glutamic Acid, D-Cellobiose, Glucose-1-Phosphate, α-Ketobutyric Acid, Phenylethylamine, α-D-Lactose, D, L-α-Glycerol Phosphate, D-Malic Acid, and Putrescine. If the microbial community could utilize the carbon source, the microplate well turned purple. The amount of utilization (how well the substrate was used) was proportional to the color intensity. Color intensity was quantified by taking the absorbance at 595 nm using a microplate reader (BioRad Model 680, Hercules, CA). Samples were measured in triplicate at 24 h, 48 h, and 72 h. The total number of used substrates (substrate richness), the best utilized substrate, and the total activity (equal to the sum of all positive absorbance values) was determined for each sample.

### Characterization of Isolates

Selected colonies grown from 14 June 2010 samples were characterized by gram-stain and by observed cell morphology (i.e., shape) using a light microscope. To obtain a diverse assortment of isolates, colonies were chosen that displayed different color plate morphologies, different colony shape and sizes, and different colony textures (e.g., shriveled, mucoid). Isolates were tested for the presence of the enzyme catalase by aseptically transferring a small amount of cells onto a glass slide and adding 2-3 drops of 3% H_2_O_2_. The observed production of bubbles was considered a positive test for catalase.

### API 20E^® ^strips

Biochemical profiles for isolates were generated using API 20E^® ^strips (bioMérieux Inc., Durham, NC). API 20E^® ^strips include enzymatic tests for fermentation or oxidation of glucose, mannitol, inositol, sorbitol, rhamnose, saccharose, melibiose, amygdalin, and arabinose, along with nitrate reduction to nitrite and nitrate reduction to nitrogen gas. API 20E^® ^strips also test for the presence of β-galactosidase, arginine dihydrolase, lysine decarboxylase, ornithine decarboxylase, citrate utilization, H_2_S production, urease, tryptophan deaminase, indole production, acetoin production (Voges - Proskauer), and gelatinase. API 20E^® ^tests were performed according to the manufacturer's instructions. The number and types of positive tests were tabulated for the isolates and used to construct biochemical phenotype profiles of the cultures which were compared amongst the isolates. A similarity dendogram among profiles was constructed using the program NTSYSpc (Exeter Software, Setauket, NY). To construct the similarity dendogram, an input matrix was constructed with the 21 API 20E^® ^tests. If a bacterial isolate was positive for that test, the matrix input was '1'. If the isolate was negative for the test, the matrix input was '0'. The NTSYSpc software was then used according to the manufacturer's instructions to produce a similarity matrix and tree. Each isolate's profile was compared to the profile of all the other isolates and reported as a Coefficient of Similarity on a scale of 0.00 - 1.00 with 1.00 equal to 100% similarity.

### Sequencing of 16S DNA

Pure cultures of bacterial isolates were identified by MIDI Labs (Newark, DE). Identification was made by sequencing approximately the first 500 bp of the 16S rRNA gene and comparing the sequences to the 16S rRNA data base by BLAST and Needleman Wunsch. Primers used were 005F (5' - TGG AGA GTT TGA TCC TGG CTC AG - 3') and 531R (5' - TAC CGC GGC TGC TGG CAC - 3'). PCR amplification and DNA sequencing was performed using the MicoSeq^® ^50016S rDNA Bacterial Identification Kits according to the manufacturer's protocols (Applied Biosystems, Foster City, CA).

### Statistical Analysis

A two-way ANOVA [29] was used to test the main effects of sample location and substrate type and their interaction on colony forming units, total activity, and number of EcoPlate™ substrates that were utilized. Significance was evaluated at α = 0.05 and when necessary, Tukey's Post Hoc tests were used to compare means within a group [30].

## Results

The measured salinity for La Sal del Rey samples (Figure 1) varied from a low of 4 ppt at site A, the water source of the lake, to a high of 420 ppt at site C just along the shoreline of the lake (Table 1). Samples from sites G and H were taken after Hurricane Alex deposited very heavy rainfall to the area, greater than 15 cm in some locations from 30 June - 2 July 2010 [31]. The salinity at site H was nearly half that measured at site C before the hurricane (Table 1).

**Table 1:** Salinity, EcoPlate™ utilization, and isolate characteristics for samples taken from La Sal del Rey 2010.

Sample	Salinity (ppt)	Preferred EcoPlate™ Substrate*	Isolates			
			
			N	Gram-negative	Rod-shaped	Percent catalase positive
**A**	4	L-phenylalanine	20	15	14	100
**B**	56	Glycogen	20^†^	16	16	95
**C**	420	Itaconic Acid	10	2	9	100
**D**	4	Tween 40	17	14	4	100
**E**	37	L-arginine	17	15	12	100
**F**	280	D-cellobiose	16	14	16	100
**G**	86	D-malic acid	nd	nd	nd	nd
**H**	220	Hydroxybutyric acid	nd	nd	nd	nd

* Highest positive EcoPlate™ absorbance value

^† ^One isolate from site B became contaminated and was eliminated from the study.

nd = not determined

Samples A - F were taken on 14 June; samples G - H were taken on 5 July after Hurricane Alex made landfall. Salinity was measured using a handheld refractometer. EcoPlates™ were inoculated with water or diluted soil samples, incubated at 25°C, and measured with a spectrophotometer (595 nm) at 24 h, 48 h and 72 h. EcoPlates™ results shown are the final results at 72 h. Isolates were selected from amongst all the samples. Some isolates contained a mixture of cell types.

**Figure 2:**
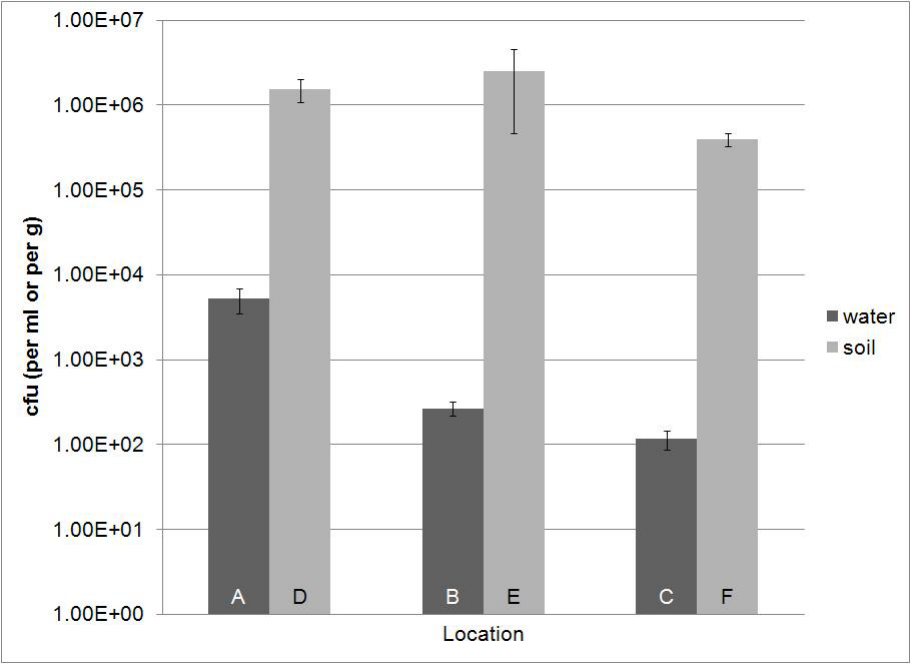
**Comparison of colony forming units per ml (cfu ml^-1^) and colony forming units per gram (cfu g^-1^) for water samples versus their counterpart soil samples taken on 14 June 2010**.

The microbial density was measured by viable plate counts on saline media containing 19.5 g L^-1 ^NaCl and other salts. Density was estimated as the number of colony forming units per ml or per gram (cfu ml^-1 ^or cfu g^-1^) for water and soil samples, respectively. Microbial density for water samples taken on 14 June 2010 (A - C) ranged from 1.2 × 10^2 ^to 5.2 × 10^3 ^cfu ml^-1^. In samples A - C, as salinity increased, the population density of the isolated species decreased (Figure 2). Soil samples taken at the same time, sites D - F, had much higher microbial densities than the water samples. After the hurricane, the cfu ml^-1 ^in water samples was substantially higher than before suggesting that salinity is a controlling factor of microbial density in the ecosystem.

Assays using EcoPlate™ microplates showed that many carbon sources could be used as substrates by the microbial communities taken from La Sal del Rey samples. The highest number of utilized substrates was observed in the community from sample G, which was positive for all 31 carbon sources tested (Table 2). The lowest number of used substrates, 12, was observed in the community in sample E. Each sample's community displayed a different preferred substrate (i.e., the greatest absorbance value) and no single substrate was preferred by all communities. The community from sample A displayed the highest total activity (the sum of all positive absorbance values) and also used a high number of substrates. The environmental conditions at site A are close to freshwater and near the water source for La Sal del Rey. The total activity was generally lower in soil samples (E - F) compared to water samples (A - C, G, H). Among water samples, the total activity was greatest at site A, decreased at higher salinities and after a hurricane event (samples G, H). Interestingly, although site A had the highest total activity, it did not have the highest microbial density whereas sites D - H had higher densities of microorganisms but not as high substrate utilization or activity.

**Table 2:** BIOLOG EcoPlate™ assays of samples from La Sal del Rey.

	Sample Location							
	**A**	**B**	**C**	**D**	**E**	**F**	**G**	**H**
**Substrate**								
β-Methyl-D-Glucoside	x	x	x	x		x	x	x
D-Galactonic Acid γ-Lactone	x	x	x	x		x	x	
L-Arginine	x	x	x	x	x	x	x	x
Pyruvic Acid Methyl Ester	x		x	x		x	x	
D-Xylose	x	x	x	x	x	x	x	
D-Galacturonic Acid	x	x	x	x		x	x	
L-Asparagine	x	x	x	x	x	x	x	
Tween 40	x	x	x	x		x	x	x
i-Erythritol	x	x	x			x	x	
2-Hydroxy Benzoic Acid			x				x	x
L-Phenylalanine	x	x	x				x	
Tween 80	x	x	x	x			x	
D-Mannitol	x	x	x			x	x	x
4-Hydroxy Benzoic Acid	x		x	x			x	
L-Serine	x	x		x	x	x	x	x
α-Cyclodextrin	x	x	x	x		x	x	
N-Acetyl-D-Glucosamine	x	x	x	x		x	x	
γ-Hydroxybutyric Acid	x	x	x	x	x	x	x	x
L-Threonine	x	x		x	x	x	x	
Glycogen	x	x		x	x	x	x	
D-Glucosaminic Acid	x	x	x	x		x	x	x
Itaconic Acid	x	x	x	x		x	x	x
Glycyl-L-Glutamic Acid	x	x	x	x	x	x	x	x
D-Cellobiose	x	x	x	x	x	x	x	x
Glucose-1-Phosphate	x	x	x	x	x	x	x	x
α-Ketobutyric Acid	x	x	x	x		x	x	
Phenylethylamine	x	x	x	x	x	x	x	x
α-D-Lactose	x	x	x	x		x	x	x
D, L-α-Glycerol Phosphate	x	x	x	x		x	x	x
D-Malic Acid	x	x	x	x		x	x	x
Putrescine	x	x	x	x	x	x	x	
**Number of Utilized Substrates**	29	28	28	27	12	27	31	16
**Total Activity**	32.858	6.995	9.492	7.780	0.397	0.768	3.794	2.506

An 'x' indicates that the organic substrate was utilized by the microbial community. Total activity is the sum of positive absorbance values for all utilized substrates.

**Figure 3:**
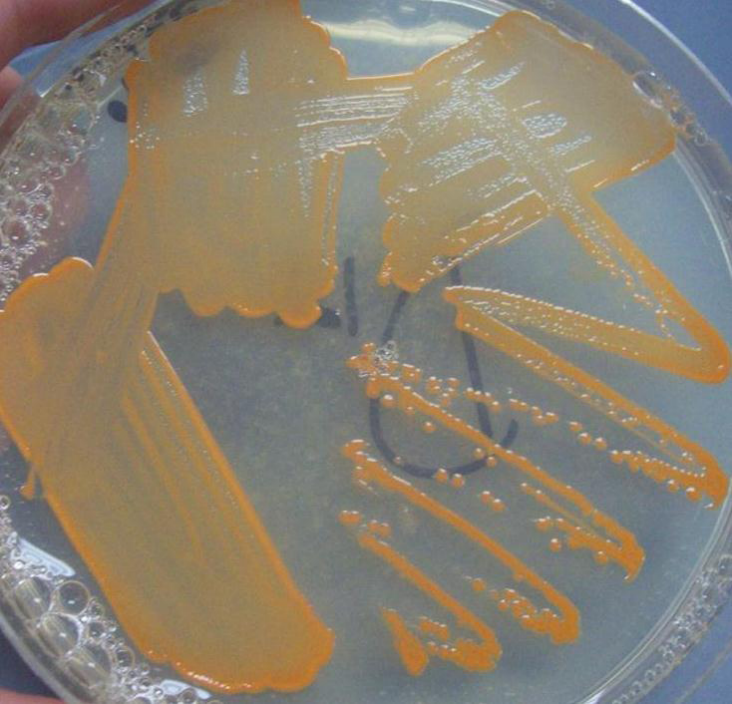
**Representative culturable halophile from La Sal del Rey, Texas**.

Random isolates were selected from agar plates inoculated on 14 June 2010. A total of 100 isolates were chosen and examined for cell morphology and the presence of the enzyme catalase. Most isolates were gram-negative (76%) and rod-shaped (71%) and virtually all were positive for catalase (Table 1). Color morphology of the cultures was typically off-white, orange-red, red, or yellow. Several cultures from site B underwent a series of color changes as the cultures aged. When these isolates were streaked onto fresh medium, the initial color of the colonies was off-white. Within 12 - 18 hours the colonies appeared green and later turned orange or orange-red (Figure 3). Further visual inspection revealed the presence of 2 strains; however, it was difficult to isolate them individually as pure cultures. When the cultures were separated, the growth of one was visibly slower and the other had very little growth. It is possible that the organisms have a symbiotic association that enhances the growth of both.

**Figure 4:**
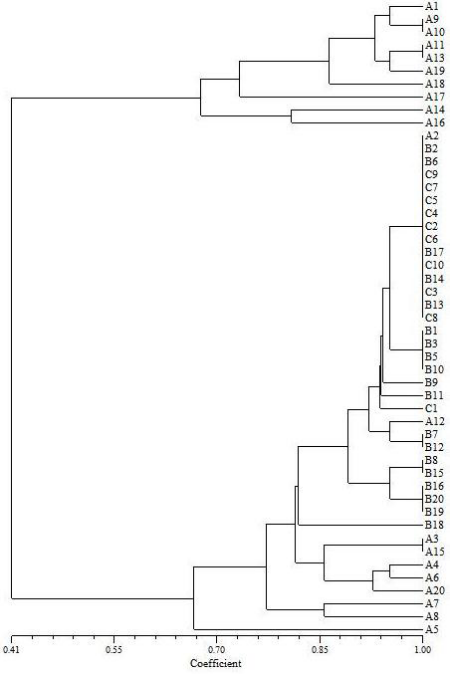
**Similarity of cultured microorganisms from La Sal del Rey based on phenotype profiles**. A total of 21 different enzymatic reactions were assayed using API 20E^® ^strips. Based on the number of positive tests and which of the 21 tests were positive, a phenotype profile was created and compared to the profile of all other tested isolates. The similarity coefficient is shown at the bottom.

Cultured isolates (n = 49) from sites A, B, and C were screened for biochemical phenotypes using API 20E^® ^test strips. A suite of 21 tests was used to construct a phenotype profile for each isolate. The number of positive tests displayed by the isolates ranged from 1 to 19. Many of the isolates that displayed only 1 positive test came from site C, which had the highest salinity (data not shown). The phenotype profiles were compared against each other and a similarity dendrogram was constructed. The similarity comparison examined both the number of positive tests and which tests were positive between organisms. Each isolate's phenotype profile was compared against the other 48 isolates' phenotype profiles. The similarity comparison is shown in Figure 4. Overall, all the isolates displayed a minimum similarity coefficient of approximately 0.4 or displayed phenotype profiles that were approximately 40% similar. The isolates' phenotypes were divided into 2 similarity clusters. A group of 10 bacterial isolates from site A, which contained the lowest salinity, formed 1 group that were > 65% similar (Figure 4, upper part of figure). A few of the isolates in that cluster showed identical phenotype profiles. Isolates A9 and A10 and isolates A11 and A13 were identical to each other. The other cluster was a mixture of isolates from sites A, B, and C. The isolates in this group generally had fewer positive tests than those isolates making up the other cluster. Many of the isolates from site C, which had the highest salinity, had identical phenotype profiles but this was generally due to those isolates displaying few positive tests. Most of the isolates from site C were only positive for a single test. The site C organisms were all positive for the Voges-Proskauer test, which is a qualitative test for the production of acetyl methylcarbinol (acetoin) from glucose. The high number and diversity of cultures from site A combined with the low number of positive tests among cultures from site C suggests that the higher salinity of the environment selects for less diversity in phenotypes. However, the change in diversity was not inversely proportional to the salinity across the entire salinity gradient.

A subset (n = 37) of the cultured organisms were presumptively identified by 16S rDNA sequencing (Table 3). All isolates were Bacteria; there were no Archaea identified. Isolate D12, although not included in the API assays, showed 95.8% similarity to *Bacillus oleronius*. Other isolates (A2, A3, A9, A12, and A16) also had the closest match to *B. oleronius*; however, the matches could not be made to the species level (i.e., a match > 99%). Furthermore, none of these isolates had identical phenotype API^® ^profiles (Figure 4). Isolates A9 and A16 were approximately 41% similar to the other isolates and were found in the same cluster on the API^® ^dendogram. Isolates A2, A3, and A12 were located in the other cluster on the dendogram and were approximately 85% similar with regards to their API^® ^profiles. The sequence and phenotype data suggests that the organisms are different species or perhaps different strains or biovars of the same species.

**Table 3:** Putative identification of isolated bacteria from La Sal de Rey, Texas, USA.

**Isolate No**.	Closest Species	Identity (%)	Confidence Level
A1	*Bacillus fastidiosis*	95.3	Genus
A2Y	*Bacillus flexus*	87.4	No match
A2D	*Bacillus oleronius*	96.1	Genus
A3	*Bacillus oleronius*	95.8	Genus
A4	*Pseudomonas fulva*	98	Genus
A7	*Bacillus megaterium*	99.9	Species
A8	*Exiguobacterium acetylicum*	92.1	No match
A9	*Bacillus oleronius*	95.3	Genus
A10	*Pseudomonas fulva*	98	Genus
A11R	*Halomonas aquamarina*	99.2	Species
A12	*Bacillus oleronius*	95.3	Genus
A13W	*Bacillus thuringiensis*	99.9	Species
A13Y	*Bacillus horikoshii*	99.1	Species
A14	*Exiguobacterium acetylicum*	92.1	No match
A15	*Bacillus firmus*	97.8	Genus
A16	*Bacillus oleronius*	95.3	Genus
A17	*Exiguobacterium acetylicum*	92.1	No match
A18	*Halomonas aquamarina*	99.1	Species
A19	*Planococcus citreus*	99.1	Species
A20	*Exiguobacterium acetylicum*	92.1	No match
B1	*Bacillus circulans*	65.4	Genus
B2	*Bacillus firmus*	97.8	Genus
B3	*Vibrio alginolyticus*	97.8	Genus
B5R	*Halomonas aquamarina*	98.7	Genus
B17	*Paenibacillus curdlanolyticus*	87.4	No match
B18	*Vibrio alginolyticus*	98.1	Genus
C1	*Bacillus firmus*	97.7	Genus
C2	*Bacillus firmus*	97.7	Genus
C3	*Bacillus firmus*	97.7	Genus
C4	*Bacillus firmus*	97.7	Genus
C5	*Bacillus firmus*	97.8	Genus
C6	*Bacillus firmus*	97.8	Genus
C7	*Bacillus firmus*	97.8	Genus
C8	*Bacillus firmus*	97.8	Genus
C9	*Bacillus firmus*	97.8	Genus
C10	*Bacillus firmus*	97.8	Genus
D12	*Bacillus oleronius*	95.8	Genus

All 10 isolates from site C, water sampled from the lake shore (salinity 420 ppt), all showed the highest 16S rDNA match to *B. firmus *(Table 3). With the exception of isolate C1, all the cultures from this highly saline site had the same phenotype profile (Figure 4). Isolates A15 and B2, from sites A and B, respectively, also showed the closest match to *B. firmus*. The confidence levels of the matches to *B. firmus *could only be made to the genus level. Sequencing of the 16S rDNA also elucidated the presence of several other potential members of the genus *Bacillus*. These included: *B. circulans*, *B. fastidiosis*, *B. megaterium*, *B. thuringiensis*, and *B. horikoshii *(Table 3).

In addition to *Bacillus*, 4 isolates (A8, A14, A17, and A20) had the closest match to the *Exiguobacterium*, which along with *Bacillus *are grouped within the Firmicutes phylum of low G+C gram-positive bacteria. Other matches to the isolates' 16S rDNA included several gram-negative organisms such as *Vibrio alginolyticus*, *Pseudomonas fulva*, and *Planococcus citreus*. Organisms A11, A18, and B5 were closely matched to the known halophile species *Halomonas aquamarina *(Table 3).

Water and soil samples A - F were taken on the same day prior to the hurricane event; therefore, we compared water samples to soil samples with regards to the cfu ml^-1 ^or g^-1^, total activity, and number of EcoPlate™ substrates that were utilized. Samples from sites G and H were not considered in the comparison because only water samples were taken on that day. The number of cfu was significantly (F_1,12 _= 13.48, P = 0.0032) higher in the soil than in the water samples (Figure 2). The overall model did not detect a significant location effect (F_2,12 _= 2.29, P = 0.1435), but this was due to the two orders of magnitude difference between the soil and water values. A secondary analysis by substrate type (ANOVA and Tukey's Post Hoc test) revealed significantly higher colony forming units in the freshwater sample than in either of the two saline samples (F_2,6 _= 25.76, P = 0.0011). No other significant effects were detected (all P > 0.1437). The amount of total activity was significantly (F_1,12 _= 196.06, P < 0.0001) higher in the water than in the soil samples and decreased significantly (F_2,12 _= 121.63, P < 0.0001) when going from fresh to salt water in both substrate types. In the water samples, total activity decreased from 32.858 in the freshwater to an average of 8.243 once the salinity was above 56 ppt. The same pattern was observed in the soil samples, but with lower activities (7.78) in the freshwater decreasing to an average of 0.583 when salinity was above 37 ppt. The number of utilized substrates was significantly (F_1,12 _= 21.17, P = 0.0006) higher in the water than in the soil samples. The water samples utilized 28.33 EcoPlate™ carbon sources on average whereas the soil samples used an average of 22.0. No other significant effects were detected (all P > 0.0681).

## Discussion

While the definition of 'halophile' varies, the traditional descriptive scheme divides halophiles into 3 categories: slight halophiles, which grow optimally at low salinities of 20-50 ppt NaCl; moderate halophiles, which grow optimally at salinities of 50-200 ppt NaCl; and extreme halophiles that grow optimally at salinities > 200 ppt NaCl [11]. The salinity of La Sal del Rey was as high as 420 ppt and soil porewater as high as 280 ppt. However, the medium used for culturing in this study contained only 19.45 ppt NaCl along with other salts. Despite the relatively low salinity of the medium used, we observed densities of approximately 10^6 ^cfu g^-1 ^for soil samples and 10^7 ^cfu ml^-1 ^or higher, especially after the passage of Hurricane Alex. The large rainfall amounts from the hurricane resulted in lower salinity of the water and may also have served as an input for nutrients into the lake, both of which may have contributed to growth of bacteria. In samples taken from 14 June 2010, prior to Hurricane Alex, we observed that soil samples contained greater densities of bacteria compared to water samples. Among water samples taken on that date, the greatest community density was observed at the lowest salinity, near the source waters of the lake. This suggests that salinity is a limiting factor in community size at least for culturable bacteria. The increase in community densities observed after the passage of the hurricane, when the salinity declined, would seem to support this. Furthermore, there was a negative correlation between the measured salinity of the sample and the estimated culturable community size.

The high density of bacteria in some samples may have been due to the presence of one or just a small number of species. The viable plate counts, API^® ^phenotype profiles, and the 16S rDNA sequencing revealed that the density and diversity of the bacterial community decreased as the salinity increased. For example, the phenotype diversity in site A (with 4 ppt salinity) was much greater than the other sites (Figure 4). Furthermore, the 16S rDNA identities of organisms from this site showed the presence of at least 5 genera and possibly 11 species (Table 2). At Site B (56 ppt), we observed 4 genera and possibly 5 species. However, at site C (420 ppt), the API^® ^phenotypes of the organisms were virtually 100% identical and the 16S rDNA sequencing showed that they were all the same genus and species. Thus, at site C the density of the organisms is most likely due to one or a few number species; at lower salinity sites, the density is greater and contains a more diverse community.

Hollister et al. [28] observed that the community diversity was not well correlated with salinity but rather was dependent on other factors such as soil water content and the amount of organic carbon in the soil. We tested the microbial communities' ability to utilize 31 different organic carbon substrates using EcoPlates™. The communities could use between 12 - 31 substrates, and again we observed a trend that the communities from samples with lower salinity could use a greater number of substrates compared to communities from samples with high salinities. Communities from water samples tended to have greater substrate utilization, both in terms of number of utilized substrates and total activity, than communities from soil samples. This was despite the typically higher cfu density in soil samples compared to water samples.

In addition to the high densities of organisms we were able to cultivate and the high percentage of carbon utilization exhibited by the community as a whole, the individual organisms we tested displayed a range of biochemical phenotypes when tested with API 20E^® ^strips. API 20E^® ^strips were designed for rapid identification of clinical organisms, especially enteric bacteria. However, many of the tests on the strip are based on traditional microbiological selective and differential media formulations or enzymatic tests. We used API 20E^® ^strips in an ecological sense in order to take the place of the traditional methods and rapidly construct a phenotype profile for the isolated organisms. The similarity dendogram that resulted from the API 20E^® ^profiles showed that although some organisms were highly similar or even 100% similar, 35% (17/49) had a unique phenotype profile. Hedi et al. [13] also used API 20E^® ^strips to characterize halophilic bacteria from a salt lake in Tunisia and observed similar results. Most of the isolates in that study were highly or 100% similar but several showed unique biochemical profiles compared to other organisms.

Hollister et al. [28] and Swan et al. [6] found high percentages of Proteobacteria in their phylogenetic analyses of La Sal del Rey and a salt lake in California, respectively. Members of the Proteobacteria are all gram-negative organisms and one subdivision in the phylum, the Gammaproteobacteria, are all rod-shaped organisms. Other studies of halophiles isolated from various habitats routinely describe gram-negative bacteria, including members of the Proteobacteria [7-9,14,32]. However, Proteobacteria are not usually the dominant phylum in hypersaline environments. Halophilic gram-positive bacteria [3], including spore-forming strains [2,13], are common. Furthermore, in some hypersaline systems Archaea dominate over Bacteria [6]. Thus, although gram-negative Proteobacteria were the dominant bacteria detected in a previous study of La Sal del Rey [28], other phyla were more easily cultured especially at lower salinities. However, this may not be the case if the experiments were repeated using buffers and media with higher salinities.

Our study suggests that molecular signatures such as those acquired for the La Sal del Rey previously [28], do not always correspond to the culturing capability of organisms from the site. Of the organisms we isolated in pure culture, most of the 16S rDNA sequences matched most closely to gram-positive genera (Table 2). This included partial matches to several species of *Bacillus *and *Exiguobacterium*. Surprisingly, some of these isolates were observed to be gram-negative rods when viewed with traditional gram staining. This initially led us to conclude that these organisms were most likely Proteobacteria. For example, isolates A9 and D12 both stained gram-negative (data not shown); however, 16S rDNA sequencing matched these organisms most closely to *Bacillus oleronius*. Kuhnigk et al. [33] isolated *B. oleronius *from the hindgut of termites and also observed that it stained gram-negative although chemical and phylogenetic assays grouped the organism with the genus *Bacillus*. The authors further observed that the isolated *B. oleronius *had a strong resemblance to other members of the genus, especially the *B. firmus*-*B. lentus *group [33]. Many of our isolates, especially those from site C, matched closely to *B. firmus*, but in many cases our sequence data only matched to the genus level. Thus, the best determination we can make to-date is that these are unknown or poorly-described members of the genus *Bacillus*, are non-obligate halophiles, and are easily cultured from La Sal del Rey.

Many of the genera and species we identified were organisms typically found in soils, such as *B. firmus*, *B. megaterium*, and *B. thuringeiensis*. The latter, *B. thuringiensis*, produces insecticidal proteins and is used in transgenic crops and to control agricultural pests [34]. Weather events, such as tropical cyclones that occur in South Texas, may contribute to the introduction of microorganisms into La Sal del Rey that may not be indigenous. The sample isolates we sequenced were collected prior to the landfall of Hurricane Alex in summer 2010; therefore, the hurricane was not a contributing factor for our sequenced isolates. The presence of these typically low G+C gram-positive soil organisms in La Sal del Rey many be due to runoff from nearby soils and fields. Inbakandan et al. [35] concluded that members of the genus *Exiguobacterium *found in marine environments probably have a terrestrial soil origin. Cultures of B. *thuringiensis *grown under hyperosmotic conditions display halotolerance [34]. Thus, many of the organisms we cultured are possibly just halotolerant and are not obligate halophiles.

Several isolated bacteria were identified as gram-negative Gammaproteobacteria (e.g., *Vibrio alginolyticus *and *Halomonas aquamarina*), which is in agreement with the culture-free studies of Hollister et al. [28]. Bacterium *V. alginolyticus *has been detected in marine and freshwaters environments and can tolerate a range of salinities [36]. Our laboratory recently identified a culture of *V. alginolyticus *from the Laguna Madre, a hypersaline estuary in South Texas, which could tolerate elevated concentrations of arsenic when grown in media supplemented with 30 ppt NaCl (Eubanks et al.: Genetic Characterization of Putative Arsenic-Oxidizing Bacteria Isolated from the Lower Laguna Madre of Texas, submitted). *Halomonas aquamarina*, also in the Gammaproteobacteria, has been described as a slight-to-moderate halophile that produces ectoine as the principle compatible solute and has been isolated from many marine environments including the Pacific Ocean, deep-sea basins, Antarctica, and hypersaline lakes [37,38]. The genus *Halomonas *has a polyphyletic evolutionary lineage and contains at least 3 main evolutionary branches [38,39]. This may explain, in part, it wide ranging distribution.

A greater density of microorganisms was observed in La Sal del Rey soil samples versus water samples. This was not surprising as soils and sediments typically have greater microbial community sizes compared to water-borne communities. Despite this, the microbial communities from water samples showed on average higher total activities and a higher number of carbon sources utilized compared to their counterpart soil samples (ABC vs DEF, respectively). This we did not expect and further demonstrates the complex nature of the microbial community inhabiting La Sal del Rey.

## Conclusions

La Sal del Rey is largely un-described with regards to the microbial ecology of the ecosystem. Traditional microbiological analysis, including culture experiments, carbon source utilization, biochemical profiling, and microscopy, along with 16S rDNA sequencing supports the hypothesis that bacteria are common organisms in the microbial community of La Sal del Rey.

## Competing interests

The authors declare that they have no competing interests.

## Authors' contributions

KP and FZ collected samples from La Sal del Rey on 14 June 2010. KP carried out the culture experiments, salinity measurements, EcoPlate™ assays, and API 20E^® ^assays. FZ participated in the design of the study, collected samples on 5 July 2010, performed all statistical analyses, and helped draft the manuscript. ORE cultivated and prepared the cultures for 16S rDNA sequencing and helped draft the manuscript. KLL conceived the study, assisted in the EcoPlate™ assays, analyzed data, and drafted the manuscript. All authors read and approved the final manuscript.
